# Rapid Determination of Non-Steroidal Anti-Inflammatory Drugs in Urine Samples after In-Matrix Derivatization and Fabric Phase Sorptive Extraction-Gas Chromatography-Mass Spectrometry Analysis

**DOI:** 10.3390/molecules27217188

**Published:** 2022-10-24

**Authors:** Bharti Jain, Rajeev Jain, Abuzar Kabir, Shweta Sharma

**Affiliations:** 1 Institute of Forensic Science & Criminology, Panjab University, Chandigarh 160014, India; 2 Central Forensic Science Laboratory, Forensic Toxicology Division, Plot #2, Sector 36-A, Dakshin Marg, Chandigarh 160036, India; 3 Department of Chemistry and Biochemistry, Florida International University, Miami, FL 33199, USA

**Keywords:** fabric phase sorptive extraction, gas chromatography-mass spectrometry, non-steroidal anti-inflammatory drugs, derivatization, urine analysis

## Abstract

Fabric phase sorptive extraction (FPSE) has become a popular sorptive-based microextraction technique for the rapid analysis of a wide variety of analytes in complex matrices. The present study describes a simple and green analytical protocol based on in-matrix methyl chloroformate (MCF) derivatization of non-steroidal anti-inflammatory (NSAID) drugs in urine samples followed by FPSE and gas chromatography-mass spectrometry (GC-MS) analysis. Use of MCF as derivatizing reagent saves substantial amounts of time, reagent and energy, and can be directly performed in aqueous samples without any sample pre-treatment. The derivatized analytes were extracted using sol–gel Carbowax 20M coated FPSE membrane and eluted in 0.5 mL of MeOH for GC-MS analysis. A chemometric design of experiment-based approach was utilized comprising a Placket–Burman design (PBD) and central composite design (CCD) for screening and optimization of significant variables of derivatization and FPSE protocol, respectively. Under optimized conditions, the proposed FPSE-GC-MS method exhibited good linearity in the range of 0.1–10 µg mL^−1^ with coefficients of determination (R^2^) in the range of 0.998–0.999. The intra-day and inter-day precisions for the proposed method were lower than <7% and <10%, respectively. The developed method has been successfully applied to the determination of NSAIDs in urine samples of patients under their medication. Finally, the green character of the proposed method was evaluated using ComplexGAPI tool. The proposed method will pave the way for simper analysis of polar drugs by FPSE-GC-MS.

## 1. Introduction

Nonsteroidal anti-inflammatory drugs (NSAIDs) are among the most frequently prescribed drugs globally. These are groups of substances with similar pharmacological profiles that include anti-inflammatory, analgesic, and antipyretic properties. Although therapeutic drugs in this class are regularly prescribed, they cannot treat the main causes of disease [1]. Despite that, these drugs are useful in reducing inflammation processes as well as the signs and symptoms of inflammatory responses. These compounds are commonly used at a growing pace since millions of individuals experience pain, whether it is acute (such as pain and fever) or chronic (such as sports injuries and arthritis) disease [2]. NSAIDs are classified into various categories based on their chemical structure (Table 7): enolic acids, pyrazolone derivatives, salicylic acid derivatives, p-aminophenol derivatives, indole indene derivatives, heteroaryl acetic acids, aryl propionic acids, anthranilic acids, and alkanones [3].

NSAIDs can inhibit arachidonic acid (AA) from being metabolized into prostaglandin (which is responsible for promoting pain, inflammation and fever) by blocking cyclooxygenase enzymes (COX-1 and COX-2). COX-1 is a structural enzyme that can produce prostaglandins, necessary for biological tissue protection [4]. On the other hand, COX-2, which is an inducible enzyme, is significantly increased in response to inflammatory cytokines or other stimuli. NSAIDs are readily absorbed after oral administration, often in the form of tablets or capsules, whereas some NSAIDs are injected into the body to prevent stomach irritability [5].

In humans, an NSAID’s biotransformation and its glucuronide metabolite are reversible to a limited extent. Nevertheless, the presence of NSAIDs in their free phase in biological samples is an indication of use at their recommended dose [6]. These compounds are regarded as safe drugs; however, their chronic abuse or overdose can also result in serious toxic side effects such as gastrointestinal bleeding, aplastic anemia, kidney failure and ulcers [7]. NSAIDs and their by-products are known to induce cellular damage in organs including the liver and kidney. Therefore, it is extremely important to establish an effective, quick, affordable and sustainable analytical method for trace level determination of NSAIDs in biological matrices.

Sample preparation plays a vital role in trace level determination of drugs in complex matrices [8]. In the past few years, various sample preparation methods have been reported for the determination of NSAIDs, such as solid-phase extraction (SPE), solid-phase microextraction (SPME), dispersive liquid–liquid microextraction (DLLME), liquid-phase microextraction (LPME), dispersive micro solid-phase extraction (DMSPE) and fabric phase sorptive extraction (FPSE) [9,10,11,12]. Additionally, new sorptive materials such as carbon nanotubes, magnetic nanoparticles, metal organic frameworks (MOFs), molecularly imprinted polymers (MIPs) and dendrimers have been synthesized and utilized in the above techniques for the extraction of NSAIDs from complex matrices [13].

Among the aforementioned sample preparation methods, FPSE is one of the most recent and robust techniques, developed in 2014 by Kabir and Furton [14]. FPSE reduces sample preparation limitations such as centrifugation, sonication, protein precipitation, and solvent evaporation. It is a novel, extremely effective, and green sample preparation method. This method comes with the benefits of sol–gel sorbent coating technology. The substrates for the FPSE microextraction device are fabrics composed of cellulose, cotton, or polyester that have been chemically functionalized with the necessary affinitive groups. Owing to the covalent bonding between the sorbent and the substrate surface in FPSE membrane, this material provides a high level of chemical, solvent, and thermal stability. Fast analyte sorption and desorption are facilitated by the open geometry of FPSE membrane, which offers high primary contact surface area. Compared to SPME, FPSE membranes possess approximately 400 times larger sorbent loading. Additionally, FPSE has the potential to tune the polarity or selectivity of the FPSE membrane by selecting the appropriate substrate, either hydrophobic or hydrophilic [15].

Among all of the contemporary microextraction techniques, FPSE has offered the maximum number of sorbents, which include polar, medium polar, non-polar, cation exchanger, anion exchanger, mixed mode, and zwitterionic multi-mode sorbents thanks to the advantages of sol–gel sorbent coating technology. Unlike classical microextraction techniques that utilize pristine organic polymers such as polydimethylsiloxane (PA), polyethylene glycol (PEG), or polyacrylate (PA), FPSE utilizes hybrid inorganic–organic polymers as the extracting sorbents. Sol–gel coating technology provides a facile pathway to combine organic polymer/inorganic polymer into a silica network. In addition, organically modified sol–gel precursors can also be used in creating the sol–gel sorbents. As such, the organic/inorganic polymer, sol–gel precursor and the surface chemistry of the fabric substrate collectively determine the overall selectivity of the FPSE membrane and can easily be fine-tuned for a given application. Extraction of analytes in FPSE membrane is primarily governed by intermolecular interactions between the analytes and the sol–gel sorbent-coated FPSE membrane. Sol–gel sorbents are inherently porous, possessing sponge-like morphology. As a result, the analytes can easily permeate through the sol–gel sorbent for successful interaction with different functional groups and interaction sites. Easy access to the interaction sites of the sol–gel sorbent results in faster extraction equilibrium.

Due to simplification of analytical workflow in sample preparation [16,17,18], a number of applications of FPSE for various analytes in a wide variety of matrices have been demonstrated, which include extraction of estrogens from urine [19], amphenicols and tetracycline from raw milk [20,21], antidepressants from urine [22], parabens from human breast tissue [23], pesticide residues from environment water and vegetables [24,25], endocrine disrupting chemicals from urine samples [26], etc.

Moreover, if analysis has to be performed by gas chromatography-mass spectrometry (GC-MS), a prior derivatization of NSAIDs is required that is usually performed by using silylation reagents. Silylation of analytes containing polar functional groups (-OH, -COOH, -NH_2_, etc.) results in replacement of their active hydrogen by a trimethylsilyl group. The derivatives thus formed are of high thermal stability and volatility [27]. However, the major drawback of derivatization with a silylation agent include the requirement of costly reagents in relatively higher amounts, lengthy reaction times (up to 60 min), anhydrous conditions and heating/microwave irradiation for the completion of reaction [28,29,30]. These drawbacks can be overcome by using alkyl chloroformate as derivatizing reagents for analytes containing polar functional groups. Derivatization with alkyl chloroformate can be performed directly in aqueous medium within a minute at room temperature [31,32,33].

Recently, green analytical chemistry (GAC) has gained prominence in sample preparation procedures. The primary goal of GAC is the development of new-generation analytical methods with the aims of minimizing reagent consumption, the possible use of biodegradable and low-toxic solvents, reduced waste generation, minimum consumption of energy, and safety to the operator/analyst along with automation and miniaturization of the analytical process. However, while meeting the criteria of GAC, it is also necessary that the developed analytical method be sufficiently sensitive, affordable and applicable in routine analysis [34].

In view of the above, the aim of the present study was to establish a green, affordable, effective and simple analytical method based on in situ methyl chloroformate (MCF) derivatization coupled with FPSE-GC-MS analysis and its application in determination of four commonly used NSAIDs (ketoprofen, ibuprofen, naproxen and diclofenac) in human urine samples. Additionally, the proposed method may open up new avenues for the simple and sensitive determination of polar analytes in biological matrices without any pre- and post-treatment using FPSE-GC-MS. Finally, the greenness of the developed method has been evaluated using prevailing indexes such as the Green Analytical Procedure Index (GAPI).

## 2. Results and Discussion

The selected NSAID drugs studied in the current project are of medium polarity to non-polar with log KOW values ranging from 3.18 to 4.51. However, sol–gel CW 20M coated sol–gel sorbent was found to be the optimum sorbent among all four tested sorbents. Although, Carbowax 20M is a highly polar polymer, the inclusion of methyl trimethoxysilane in the sol solution makes the resulting sorbent highly affinitive towards both polar, medium-polar and non-polar sorbents. The sol–gel CW20M provides different intermolecular interactions including dipole–dipole interaction, hydrogen bonding, and London dispersion. 

### 2.1. Screening of FPSE Membrane

**Figure 1:**
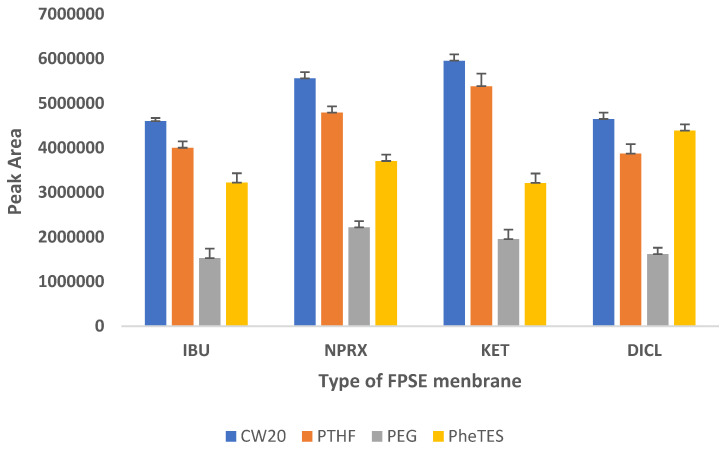
Screening of different FPSE membranes for NSAIDs.

In order to select the most efficient sorbent chemistry that was compatible with the target analytes in terms of selectivity and extraction efficiency, four different FPSE substrates coated with sol–gel phenyl triethoxysilane (sol–gel PheTES) (polar), sol–gel polyethylene glycol 10,000 (sol–gel PEG 10,000) (polar), sol–gel polytetrahydrofuran (sol–gel PTHF) (medium polar) and sol–gel Carbowax 20M (sol–gel CW20M) (polar) were assessed for the extraction of NSAIDs from urine samples using the peak area as evaluation parameter. The findings, which are depicted in Figure 1, revealed that NSAIDs are best extracted by sol–gel Carbowax 20M, followed by sol–gel PTHF, sol–gel PheTES, and sol–gel PEG. Therefore, sol–gel CW20M was used as the sorptive phase for further experiments. 

### 2.2. Screening of Back-Extraction Solvent

Analytes should be back-extracted for further chromatographic analysis when they are efficiently extracted with FPSE membrane. For this purpose, three different solvents, MeOH, EtOH and ACN along with mixtures of two solvents such as MeOH:ACN and EtOH:ACN (50:50 *v*/*v*) were studied. As illustrated in Figure S1, MeOH was the best solvent to back-extract the NSAIDs from FPSE membrane. Therefore, MeOH was used as the back-extraction solvent for all further experiments.

### 2.3. Optimization of Derivatization Conditions

MCF easily interacts with analytes that have polar functional groups such as phenolic alcohol, carboxylic acid and amines. MCF produces carbonate with phenols, whereas carboxylic acid is first transformed into anhydride and then methyl ester via decarboxylation. PYR acts as an acid scavenger as well as catalyst in the reaction. In order to obtain highest derivatization efficiency of MCF for NSAIDs, the volumes of MCF and pyridine were studied. A set of experiments was carried out in which 10 mL of diluted urine sample in a 15 mL of glass vial was spiked with 10 μg mL^−1^ of NSAIDs. To each glass vial, PYR was added in a range from 50–200 μL. A constant amount of MCF, i.e., 50 µL was added to each sample, and the reaction was carried out at room temperature for 30 s. Subsequently, the FPSE membrane was immersed into the sample and placed on a magnetic stirrer at 1000 rpm for 30 min. After extraction of derivatives of NSAIDs, the membrane was taken out and immersed into a vial containing 500 μL of MeOH for 10 min to back-extract the analyte and analyzed by GC-MS. From Figure S2a, it is evident that peak areas for NSAIDs increased from 50 to 100 μL and tended to decline with further increases in the volume of PYR. Hence, a volume of 100 μL of pyridine was chosen for further experiments. To examine the impact of MCF volume on derivatization efficacy, four distinct volumes were studied, ranging from 50–200 μL. A constant volume of 100 μL of pyridine as finalized in the previous experiment was added to the sample. All other experimental conditions were identical to the previous experiment. As depicted in Figure S2b, the peak area of 50 μL of MCF was the highest among all tested volumes. Therefore, 50 μL of MCF was selected for further experiments.

### 2.4. Multivariate Optimization

#### 2.4.1. Plackett–Burman Design (PBD)

The current study was designed to screen the significant factors based on their major effects on the extraction efficiency of FPSE rather than the interaction effects between multiple factors; thus, the Plackett–Burman design was used [35]. Herein, seven independent factors were evaluated with maximum (+1) and minimum (−1) levels. As can be seen in Table 1, these independent factors were: (i) volume of sample (mL), (ii) extraction speed (rpm), (iii) extraction time (min), (iv) ionic strength (%), (v) pH, (vi) elution time (min), and (vii) derivatization time (min). For the identification of significant variables, a 2^7−4^ PBD was used. All of the experiments were carried out in triplicate and in a random manner for 24 runs (7 + 1 = 8 ∗ 3 = 24). An analysis of variance (ANOVA) test was used to analyze the significant factors. If the confidence level of the parameter was more than 95%, at that moment the significance level rose to *p*< 0.05. The variables with a confidence level greater than 95% were considered significant for inclusion in further optimization experiments. A standardized Pareto chart depicted the findings of PBD experiments (Figure S3). The bars were arranged according to the sizes of the effects, with the major one at the top. As illustrated in Figure S3, variables such as pH, extraction time, derivatization time, sample volume, ionic strength, elution time, and extraction speed indicating *p*-values of less than 0.05 were found to have a significant effect on the extraction efficiency of FPSE. According to Figure S3, the following factors were listed in order of significance: pH > extraction time > derivatization time > volume of sample > ionic strength > elution time > extraction speed. Among all of these significant factors, derivatization time and volume of sample had a greater impact on extraction efficiency of FPSE, followed by ionic strength, elution time and extraction speed. In view of this, the most significant factors such as pH, extraction time and derivatization time were chosen for subsequent optimization experiments.

#### 2.4.2. Central Composite Design (CCD)

In order to optimize the most significant variables from PBD experiments, a CCD based on response surface methodology (RSM) was applied. Five different levels of the above-mentioned most significant factors were used to analyze each of the independent factors, i.e., factorial points (−1, +1), axial points (−α, +α), and central point (0). The total number of experimental runs was calculated using the equation appended below:

N = 2^k^ + 2K + Cp

where k = number of factors and Cp = number of center points. In this manner, 19 runs were performed with 8 factorial points (2^k^), 6 axial points (2K), and 5 center points. The parameters and their levels as well as the CCD matrix are listed in Table 2 and Table 3. For CCD experiments, the cumulative peak area of all analytes was used as the response. To identify the individual significant effects and the interactions between the significant parameters, analysis of variance (ANOVA) was carried out on the data.

**Figure 2:**
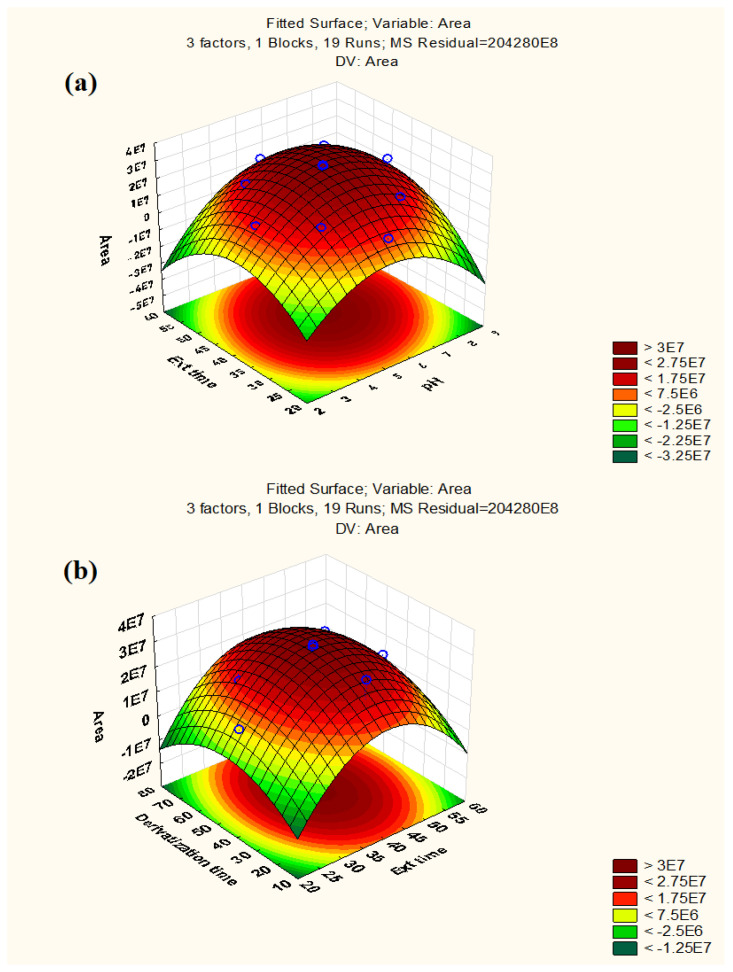
Response surface plots: (**a**) Extraction time vs. pH (**b**) Derivatization time vs. Extraction time.

To determine the significant interactions between the parameters, three-dimensional response surface plots were used. Figure 2 displays the response surface graphs, which demonstrate the interactions between extraction time and pH and between derivatization time and extraction time, respectively, keeping the third factor constant at its central level. According to response surface graphs, the maximum response for extraction time was obtained in the range of 40–45 min, whereas the highest response for pH was between 5–7. Similarly, the optimum response for derivatization time was found to be 40–45 s. As seen in Figure S4, the desirability profile (DP) offered the actual optimum value for all of these parameters. The RSM results were converted into a scale of desirability in DP, ranging from totally undesirable (0) to fully desirable (1). A series of graphs were formed for each independent parameter, and a red line showed the final optimal value on a graph. The ideal values for these parameters were: 45 s (derivatization time), 40 min (extraction time), and 5.5 (pH). For experimental purposes, the value of pH was rounded off to 6. 

#### 2.4.3. Analytical Performance of the Method

Under the optimized conditions, the proposed FPSE-GC-MS method for determination of NSAIDs was validated for its linearity, precision, recovery, LODs, and LOQs according to international guidelines (USFDA, ICH). Urine samples were spiked with the target analytes at different concentrations in the range of 0.1 –10 μg mL^−1^. Correlation coefficient, slope and intercept were calculated by least squares linear regression analysis. Linearity was calculated by plotting the peak area ratio of analyte and internal standard on the *y* axis and corresponding concentration on the *x* axis. The concentration and peak area ratios of analyte and IS of all tested drugs were found to be highly correlated (0.998–0.999) in the linear range of 0.1–10 µg mL^−1^. Signal-to-noise ratios of 3:1 and 10:1 were used to calculate the LOD and LOQ, respectively. The proposed method has been found to be adequately sensitive for the identification of target analytes in human urine as revealed by the LOD and LOQ being in the range of 0.0015–0.0049 and 0.0049–0.016 μg mL^−1^, respectively. Furthermore, repeatability and reproducibility of the assay were evaluated in terms of intra-day and inter-day precisions, which were expressed as %RSD. Intra-day precisions were calculated at low, middle and high QC concentrations (*n* = 5), whereas inter-day precisions were calculated by analyzing the same concentrations over the course of five consecutive days. Percent RSDs for intra-day and inter-day precisions were less than 7 and 10%, respectively, as shown in Table 4. Additionally, recoveries were calculated by comparing the concentration found and nominal concentration of the analyte in spiked urine samples. The absolute and relative recovery values ranged from 86.6–113.1 and 87.6–98.9, respectively, for all tested NSAIDs (Table 5).

The proposed method has been compared with previously published methods for determination of similar analytes from different matrices, which are shown in Table 6. It can be observed from Table 6 that the method reported herein offered comparable precision with other sample preparation protocols. Sensitivity of the proposed method was fit for the purpose as it was able to detect low concentrations of drugs in real human urine samples from two subjects who were under NSAID medications. It must be highlighted that the proposed method utilizes only 0.5 mL of MeOH for back-extraction, and no other solvent is consumed during the process. Moreover, no additional step of centrifugation and/or vortex agitation was required, as in Refs. [36,37,38,39,40]. Although the SPE-GC-MS [36] and DLLME-GCMS [37] methods have lower LODs than the proposed method for similar analytes, SPE involves multistep operation and requires large solvent volumes for eluting retained analyte from the SPE bed. The derivatization of extract was performed using BSTFA+TMCS under microwave irradiation for 3 min at 350 watts [36]. In DLLME-GC-MS [37], 1 mL of acetone and 0.25 mL of dichloromethane were used as disperser and extraction solvent, respectively. Following the DLLME process, the extract was submitted for derivatization with BSTFA+TMCS at 50 °C for 30 min. The proposed method has clear-cut operational advantages over the SPE and DLLME methods, such as: (i) FPSE involves a limited number of sample preparation steps, thus eliminating the errors generated and consumption of only 0.5 mL of MeOH in the whole analytical procedure, and (ii) in-matrix derivatization using MCF does not require any external heating conditions, prolonged reaction time, moisture-free atmosphere and microwave irradiation.

#### 2.4.4. Evaluation of Green Character of the Proposed Method

**Figure 3:**
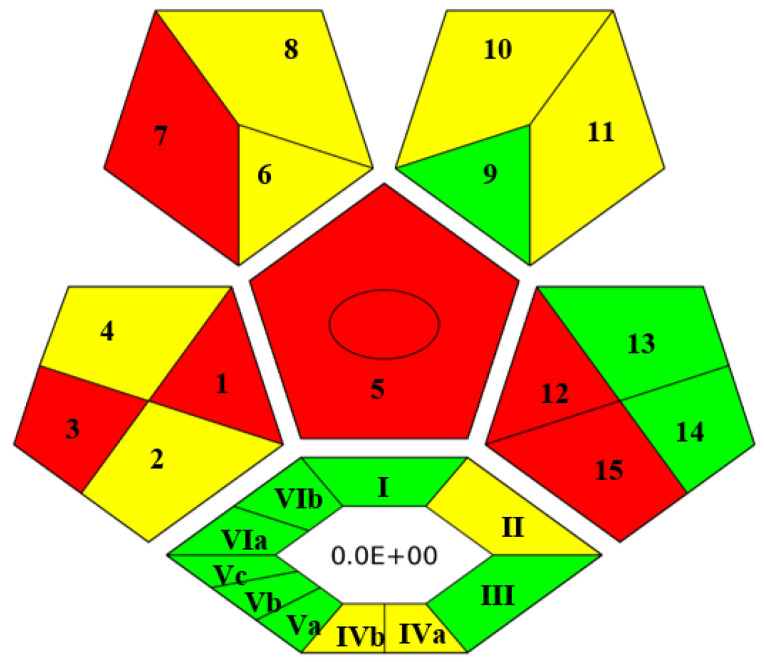
ComplexGAPI pictogram for proposed method.

In recent years, several metrics to assess the greenness of analytical methods have been developed. Among them, the Complex Green Analytical Procedure Index (ComplexGAPI) is the most comprehensive and becoming very popular nowadays [41]. As a smart tool, ComplexGAPI was used to evaluate the greenness of the developed method. Upon entering values in different parameters of GAPI software as shown in Figure S5, a pictogram was obtained, composed of five pentagrams and one hexagon at its bottom. The color (red, yellow and green) of each pentagram describes the degree of impact of the concerned analytical step on the environment. In this way, the final GAPI pictogram depicts a comprehensive and quick outlook for the greenness of the analytical method. The hexagon at the bottom of the pictogram depicts the environmental impact of pre-analytical processes. The pictogram of ComplexGAPI for the proposed analytical method is shown in Figure 3. From Figure 3, it can be claimed that the proposed analytical method is sufficiently green and does not impose any adverse effects on the environment, as most of the pentagrams are either yellow or green, which means adherence to the principles of GAC. The red pentagrams were 1, 3, 5, 7, 12 and 15 (Figure 3), which corresponded to sample collection (off-line), transportation (required), extraction (required), solvent used (non-green, i.e., MeOH), energy consumption (>1.5 kWh/hour for GC-MS) and waste treatment (no treatment), respectively. 

### 2.5. Application to Real Samples

**Figure 4:**
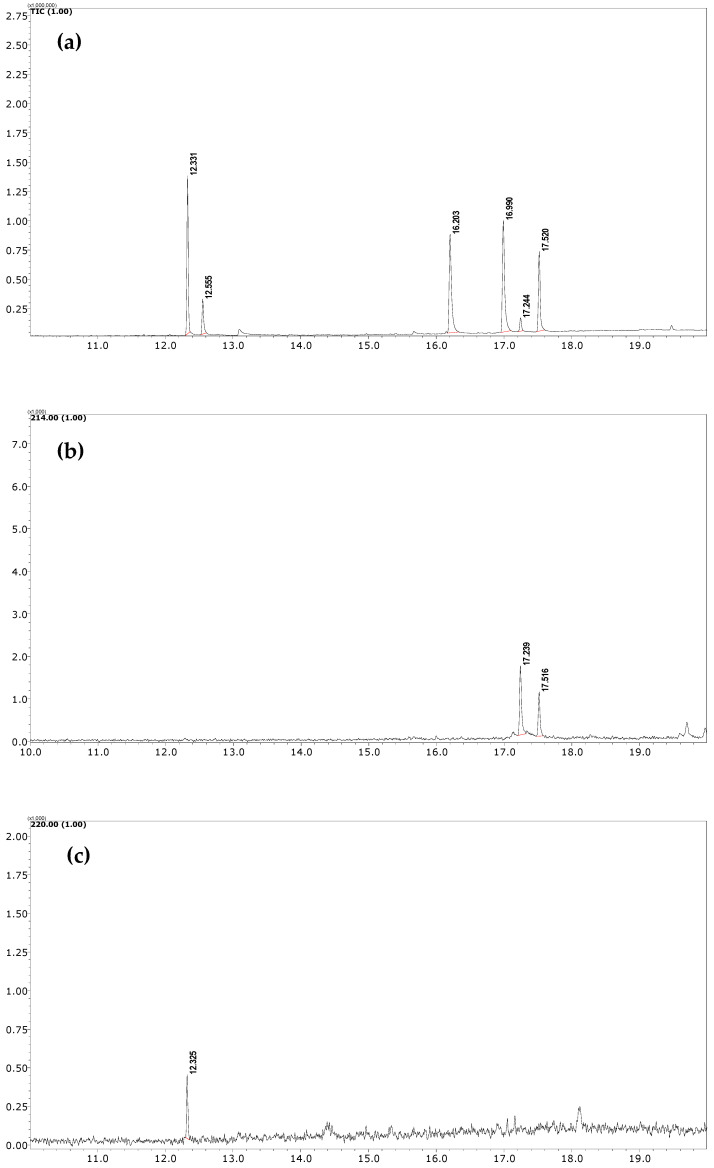
(**a**) TIC of standard sample at 10 µg mL^−1^ (**b**) SIM chromatogram of urine sample of donor who had consumed DICL; (**c**) SIM chromatogram of urine sample of donor who had consumed IBU.

The developed FPSE-GC-MS method has been successfully applied to quantify NSAIDs in human urine samples under optimum conditions. Male and female participants aged 30–40 years who were under NSAID medications (DICL and IBU, 100 mg each) donated their urine samples after 4–6 h of consuming the respective tablets. Samples were stored at ~4 °C until analysis. GC-MS chromatograms of these samples after the FPSE procedure are shown in Figure 4. It is worth noting here that an additional peak (at 17.24 min), just near the peak of DICL (RT = 17.51 min), was observed in the GC-MS chromatogram due to formation of an artifact under acidic conditions during the extraction process and was identified as 1-(2,6-dichlorophenyl) indolin-2-one by the NIST library. Therefore, as suggested in [42], we added the peak area of this artifact in the final quantification of DICL. In real samples, the concentration levels of DICL and IBU were found to be 0.122 and 0.04 µg mL^−1^, respectively.

## 3. Experimental Section

### 3.1. Materials and Methods

The Department of Chemistry and Biochemistry at Florida International University in Miami, Florida (USA) synthesized and characterized sol–gel sorbent-coated FPSE membranes. Joanne Fabrics supplied unbleached cotton fabric that was 100% cellulose (Miami, FL, USA). Standards of diclofenac (DCL), ibuprofen (IBU), naproxen (NAP), ketoprofen (KET), and pregabalin (PRG) were obtained from Indian Pharmacopoeia Commission (IPC, Ghaziabad, India). PRG was used as an internal standard (IS). HPLC-grade methanol (MeOH, purity > 99%), acetonitrile (ACN, purity > 99%), and acetone (ACE) were obtained from Thermo Fisher Scientific (Waltham, MA, USA). Sodium chloride (NaCl) (purity > 99%), Poly(tetrahydrofuran) (PTHF), Phenyl triethoxysilane (PheTES), methyltrimethoxysilane (MTMS), Carbowax 20M (CW 20M), Poly (ethylene glycol 10,000) and trifluoroacetic acid were procured from Sigma-Aldrich (St. Louis, MO, USA). Polyethylene glycol (PEG) was obtained from Alfa Aesar (Ward Hill, MA). Methyl chloroformate (MCF, derivatizing reagent) and pyridine (PYR) were obtained from Sigma-Aldrich, Switzerland. A Milli-Q water purification system (Millipore, Bradford, MA, USA) was used to produce ultrapure water. All of the chemicals and reagents used in this study were of analytical grade unless otherwise stated.

### 3.2. Preparation of Standards and Samples

Stock solutions of NSAIDs were prepared at a concentration of 1 mg mL^−1^ by dissolving their respective standards in MeOH. The stock solutions were maintained at ~4 °C. Blank and real urine samples were obtained from authors of this study aged between 30–40 years on medication of NSAIDs with prior consent. Blank urine samples fortified with NSAIDs were utilized for method development and validation studies without any pre-treatment, e.g., filtration, centrifugation. Stock solution of PRG (internal standard) was also prepared at 1 mg mL^−1^ in MeOH. Quality control (QC) samples were prepared at three different concentration levels of calibration graph, i.e., 0.3 µg mL^−1^ (low QC), 1 µg mL^−1^ (medium QC), and 10 µg mL^−1^ (high QC).

### 3.3. Fabrication of FPSE Membrane 

The current project involved creation of four different sol–gel sorbent-coated FPSE membranes using unbleached cotton, 100% cellulose fabric as the substrate. The selected sol–gel sorbents included sol–gel phenyl triethoxysilane (sol–gel PTES), sol–gel poly(ethylene glycol) 10,000 (sol–gel PEG 10,000), sol–gel poly(tetrahydrofuran) (sol–gel PTHF) and sol–gel Carbowax 20M (sol–gel CW20M). The sol solutions for the sol–gel sorbent coatings on cellulose fabric substrate were prepared independently using optimized formulation. The sol solution was prepared by sequential addition of 10 mL sol–gel precursor methyltrimethoxysilane (MTMS), 10 mL methylene chloride, 10 mL acetone, 5 g polymer/precursor and 4 mL aqueous trifluoroacetic acid (95% in water) into a large centrifuge. The composite mixtures were vortexed for 3 min, centrifuged for 5 min, and sonicated for 2 min, and the clear supernatant parts of the sol solutions were finally transferred to clean 89 mL amber colored glass reaction bottles. The cellulose fabric substrates during the sol–gel coating process were kept submerged into the sol solutions for 4 h to form the sol–gel coating around the micro fibrils of the substrate. 

Following the completion of the residence time of the cellulose fabric substrates inside the sol solution, the coated substrates were removed from the sol solutions and were kept in the desiccator overnight for the evaporation of the solvent and aging of the sol–gel sorbent coating. The coated FPSE membranes were subsequently rinsed with methylene chloride:acetone (50:50; *v*/*v*) under sonication for 30 min to remove unreacted and unbonded residual sol solution ingredients from the coated surface. The FPSE membranes were then air dried for 1 h and stored in airtight containers to prevent accumulation of unwanted analytes from the environment. The sol–gel sorbent-coated FPSE membranes were then cut into the required size of 1 cm. × 1.5 cm.

### 3.4. In-matrix Derivatization and FPSE Procedure

**Figure 5:**
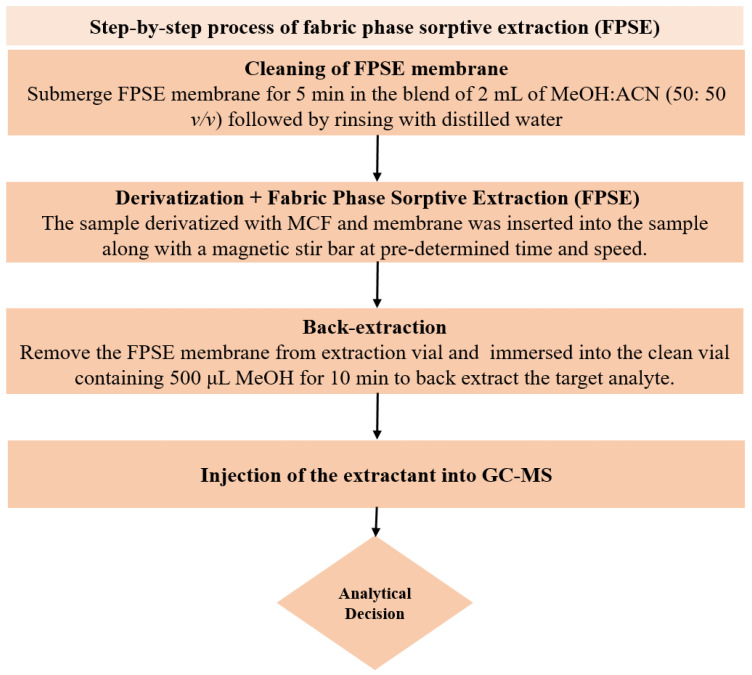
Schematic depiction of the steps included in FPSE procedure.

In order to clean the FPSE membrane, the sol–gel CW 20M coated FPSE membrane was initially submerged in the mixture of 2 mL of MeOH and ACN (50: 50 *v*/*v*) using tweezers (to prevent contaminations from contact) for 5 min. Cleaned FPSE membrane thus obtained was then rinsed with 2 mL of deionized water to eliminate any remaining organic solvents before being immersed into the sample for extraction. One mL of urine sample containing analytes and IS at 1 µg mL^−1^ was then diluted up to 10 mL with ultrapure water in a 15 mL glass vial, and the pH was adjusted to 6. For in situ derivatization of NSAIDs; 50 μL of PYR and 50 μL MCF were added in the sample and gently agitated using a vortex shaker for 45 s. At this stage, all of the NSAIDs were derivatized into their corresponding methyl esters at room temperature directly in the aqueous medium. Next, the cleaned FPSE membrane was inserted into this sample vial and placed on a magnetic stirrer for extraction of NSAIDs at 1000 rpm for 30 min. After extraction, the membrane was taken out with the help of tweezers and then immersed into an Eppendorf tube containing 500 μL MeOH for 10 min to back-extract the target analyte without any additional step of vortex, centrifugation or sonication. Now, this eluant was ready for GC-MS analysis. The membrane could be reused by rinsing with a solvent system of 2 mL of MeOH and ACN (50:50 *v*/*v*) for 5 min. The systematic FPSE protocol is depicted in Figure 5.

### 3.5. Gas Chromatography-Mass Spectrometric (GC-MS) Analysis

Analysis of the extract obtained after performing the FPSE procedure was performed on a Shimadzu Nexis GC-2030 hyphenated with QP-2020 NX mass spectrometer. The GC-MS system was equipped with an AOC-20i auto injector. Exactly 2 µL of extract was injected at 250 °C (split value 10) into the GC-MS injection port, which was connected with an SH-Tri-5Sil MS capillary column (30 m length × 0.25 mm internal diameter × 0.25 µm film thickness with stationary phase of 5% phenyl and 95% dimethylpolysiloxane). Helium was used as carrier gas at a flow rate of 1 mL min^−1^. The oven temperature was initially kept at 70 °C for 4 min and gradually increased up to 280 °C at a rate of 15 °C/min and finally kept at that temperature for 2 min, resulting in a total run time of 20 min. The temperatures of transfer line and ion source were set at 250 and 200 °C, respectively. Ionization of analytes was performed at an electron energy of 70 eV in positive ionization (+EI) mode. Initially, for identification purposes, the mass spectra of analytes were recorded in full scan mode (50–500 amu). For further studies, analytes were quantified in selected ion monitoring (SIM) mode. The retention times, selected ions, molecular weights (before and after derivatization) and structures of studied NSAIDs are depicted in Table 7.

### 3.6. Multivariate Optimization

We examined various experimental parameters that can influence the extraction efficacy of FPSE, including sample volume, ionic strength, pH, extraction time, extraction speed, derivatization time and elution time. For this purpose, multivariate analysis was carried out to study these parameters in two segments viz. (1) Plackett–Burman design (PBD) to screen the significant parameters and (2) a central composite design (CCD) to optimize the significant parameters obtained by PBD. With the aid of the TIBCO STATISTICA software (Palo Alto, CA, USA, Trial version), statistical analysis was performed.

### 3.7. Method Validation

In terms of accuracy, sensitivity, linearity, and precision, the proposed analytical method was validated. The linearity of the proposed method was studied by plotting matrix-matched calibration curves directly in fortified urine samples in the range of 0.1–10 µg mL^−1^. The slope, intercept, and coefficient of determination were determined using linear regression analysis. Signal to noise (S/N) ratios of 3.3 and 10 were used to calculate the limits of detection (LOD) and quantification (LOQ), respectively. The determination of intra-day and inter-day precisions was based on the evaluation of the relative standard deviation (%RSD) at three different concentration levels of calibration graph. The absolute and relative recoveries were evaluated at three different concentrations i.e., 0.3 µg mL^−1^ (low QC), 1 µg mL^−1^ (medium QC) and 10 µg mL^−1^ (high QC) 

## 4. Conclusions

In the present study, for the first time, in-matrix alkyl chloroformate derivatization was explored as a simple, rapid and effective derivatization approach in combination with FPSE-GC-MS analysis for determination of four commonly used NSAIDs (KET, IBU, NAP, and DICL) in human urine samples. In comparison to conventional silylation, the use of MCF as derivatizing reagent saves a significant amount of time, reagent and energy and can be directly performed in aqueous samples without any sample pre-treatment. Additionally, derivatized analytes can be conveniently extracted by FPSE membrane and analyzed by GC-MS, which effectively eliminates sample reconstitution, solvent evaporation, filtration, and centrifugation from the sample preparation framework. In the whole process, only 0.5 mL of MeOH is consumed, which significantly reduces the consumption of organic solvents to satisfy the requirements outlined by Green Analytical Chemistry (GAC) principles. FPSE is quick and needs only a few sample preparation steps, which significantly reduces the likelihood of error. A strong chemical bond between the sorbent and the substrate contributes to the high chemical and solvent stability of FPSE media, which also allows any organic solvent to be used as the eluant for the analyte’s back-extraction. This enables the analysis of the same sample in multiple chromatographic techniques. In conclusion, the present method can be routinely employed for analysis of NSAIDs for therapeutical drug monitoring purposes as well as in clinical and forensic toxicological laboratories. This approach will also pave the way for the development of novel analytical methods for polar analytes using the FPSE-GC-MS module.

## Figures and Tables

**Table 1:** Levels of independent variables for PBD design.

Factors	Levels	
	Low (−1)	High (+1)
Volume of sample (mL)	5	10
Ionic strength	0	10
pH	5	8
Extraction time (min)	30	60
Extraction speed (rpm)	500	1000
Elution time (min)	5	15
Derivatization time (s)	30	60

**Table 2:** Significant factors and their levels studied by CCD.

Factors	Levels			Star points	
	Low (−1)	Central (0)	High (+1)	−α	+α
pH	4	5.5	7	2.977	8.02
Extraction time (min)	30	40	50	23.18	56.81
Derivatization time (s)	30	45	60	19.77	70.22

**Table 3:** Experimental design of CCD.

Runs	pH	Extraction Speed (RPM)	Volume of Sample (ML)
1	−1	−1	−1
2	−1	−1	+1
3	−1	+1	−1
4	−1	+1	+1
5	+1	−1	−1
6	+1	−1	+1
7	+1	−1	−1
8	+1	+1	+1
9	-α	−1	0
10	+α	−1	0
11	0	−α	0
12	0	+α	0
13	0	0	−α
14	0	0	+α
15	0	0	0
16	0	0	0
17	0	0	0
18	0	0	0
19	0	0	0

**Table 4:** Analytical characteristics of FPSE-GC-MS method for NSAIDs.

Drug	LOD (µg mL^−1^)	LOQ (µg mL^−1^)	R^2^	Linearity (µg mL^−1^)	Calibration Curve	Precision (%RSD)					
						Intra-Day (µg mL^−1^)			Inter-Day (µg mL^−1^)		
						0.3	1	10	0.3	1	10
DIC	0.0049	0.016	0.998	0.1–10	y = (17,062 ± 297.6)x + (29,015 ± 1365.9)	5.2	4.1	3.1	6.2	8.1	9.2
IBU	0.0022	0.0072	0.999	0.1–10	y = (35,576 ± 447.8)x + (22,877 ± 2055.1)	3.8	6.2	5.8	8.0	5.4	6.5
NAP	0.0015	0.0049	0.999	0.1–10	y = (43,090± 429.5)x + (41,269 ± 1971.3)	4.6	3.7	2.4	9.5	7.1	5.6
KET	0.0031	0.0103	0.999	0.1–10	y = (13,066 ± 128.6)x + (28,573 ± 590.3)	6.2	4.9	3.5	7.5	6.4	8.8

**Table 5:** Extraction efficiency parameters of proposed method.

Drug	RR%			Absolute Recovery (%)		
	0.3 µg mL^−1^	1 µg mL^−1^	10 µg mL^−1^	0.3 µg mL^−1^	1 µg mL^−1^	10 µg mL^−1^
DIC	90.1	96.2	98.2	86.6	95.6	98.2
IBU	92.5	89.5	97.5	108.1	113.1	100.6
NAP	87.6	94.4	96	109.4	112.2	100.2
KET	92.9	96.7	98.9	99.8	91.8	99.3

**Table 6:** Comparison of the proposed method with previously published methods for determining NSAID drugs in different matrices.

Sr. No.	Method	Matrix	Analyte	LOD (ng mL^−1^)	LOQ (ng mL^−1^)	Linearity Range (ng mL^−1^)	Precision	Ref.
1	SPE-GC-MS	Urine and Blood	ASA, Carbamazepine, Chloramphenicol, Clofibric acid, DIC, 17α-ethinylestradiol, 17β-estradiol, Estrone, Florfenicol, Flunixin, IBU, KET, MFA, Metoprolol, NAR, NFA, APAP, Phenyl butazone, Propranolol, Pyrimethaimne, Thiamphenicol, Triclosan	0.0002–0.0013 (blood) 0.0008–0.0056 (urine)	NA	0.0006–5	<7.5%	36
2	DLLME-GCMS	Animal Urine	ASA, IBU, NAP, KET	0.1–4.1	0.2–4.7	1–100	<5%	37
3	LLE-GC-MS	Human Plasma	NAP	30	100	100–5000	5.14%	38
4	ASE-SPE-GCMS	Tissue	IBU, APAP, DIC, NAR, KET, and three estrogens	1000–7000	3000–22,000	19,500–25,000,000	<5%	39
5	LLE-GC-MS	Human Serum	DCF, APAP, IBP, NI, NFA, MFA, NAR, ASA, SA	2–124	6–414	200–1,000,000	<5%	40
6	FPSE-GC-MS	Environment Water	IBU, NAR, KET, and DIC	0.8–5	3–15	5–500	<5%	28
7	FPSE GC-MS	Human Urine	IBU, NAR, KET, and DIC	1.5–4.9	4.9–16	100–1000	<7%	Present study

APAP—Acetaminophen; ASA—Acetylsalicylic acid; SA—Salicylic acid; IBU—Ibuprofen; DIC—Diclofenac; NI—Nimesulide; NFA—Niflumic acid; MFA—Mefenamic acid; NAR—Naproxen; KET—Ketoprofen.

**Table 7:** Selected Physicochemical properties of the analytes, GC–MS retention times and prominent ions.

Drug	Molecular Weight (before Derivatization)	Retention Time (min)	Selected Ion *(m*/*z)*	Structure of Polar Drugs (after Derivatization)
Ibuprofen	206.28	12.36	161, 220, 117	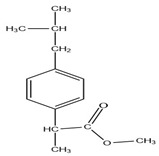
Ketoprofen	254.28	17.01	209, 105, 268	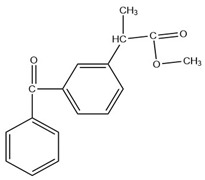
Diclofenac	296.1	17.55	214, 242, 304	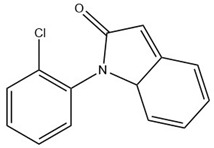
Naproxen	230.26	16.23	185, 244	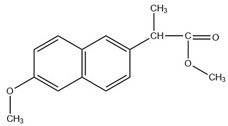
Pregabalin	159.23	12.57	88,158,114	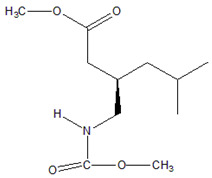

## Data Availability

Data available on reasonable request.

## Supplementary Materials

The following supporting information can be downloaded at: https://www.mdpi.com/article/10.3390/molecules27217188/s1, Figure S1: Screening of back-extraction solvent on extraction efficiency of FPSE; Figure S2: Optimization of derivatizing reagent for NASIDs: (a) volume of pyridine (μL) (b) volume of MCF (μL); Figure S3: Pareto diagram of calculated main effect for PBD; Figure S4: Desirability function plot showing the optimum range of significant factors; Figure S5: Input parameters and their values in ComplexGAPI software for the proposed method.
