# Supplementary material for: Rapid Determination of Non-Steroidal Anti-Inflammatory Drugs in Urine Samples after In-Matrix Derivatization and Fabric Phase Sorptive Extraction-Gas Chromatography-Mass Spectrometry Analysis
Source: Molecules. 2022 Oct 24;27(21):7188. doi: 10.3390/molecules27217188 (PMC9657276; doi:10.3390/molecules27217188)
Supplement: Supplementary file 1 [file molecules-27-07188-s001.zip › molecules-1955479-supplementary-done.pdf]

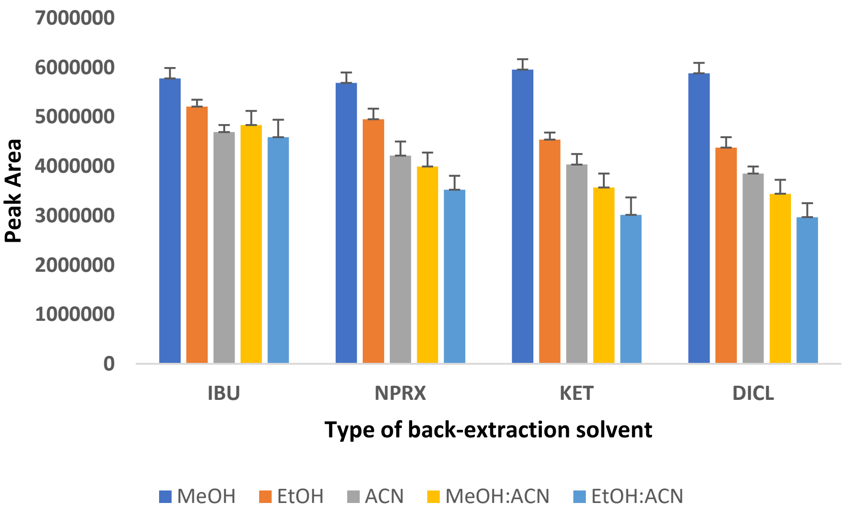

Figure S1. Screening of back-extraction solvent on extraction efficiency of FPSE.

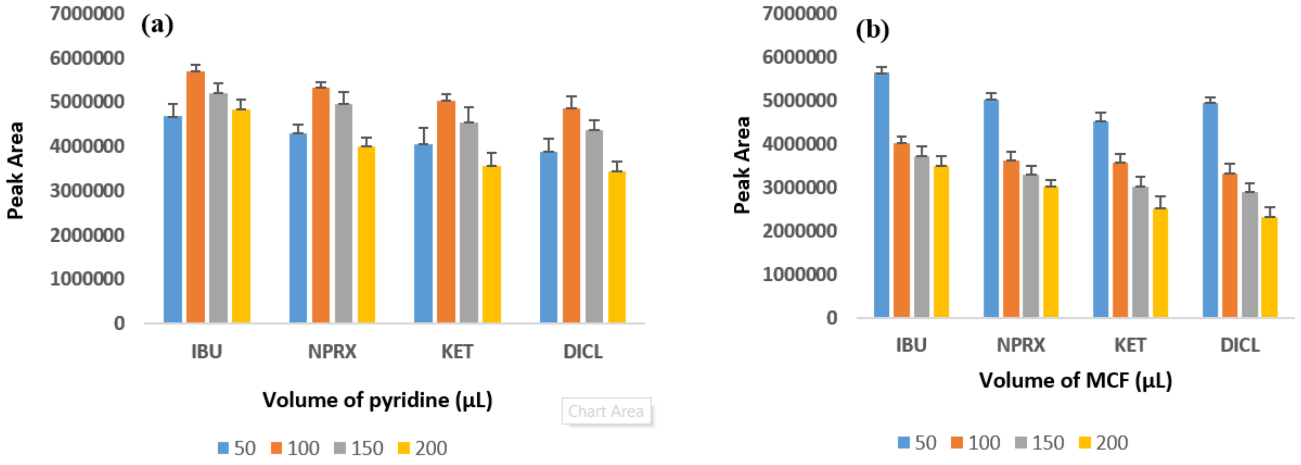

Figure S2. Optimization of derivatizing reagent for NASIDs: (**a**) volume of pyridine (μL) (**b**) volume of MCF (μL).

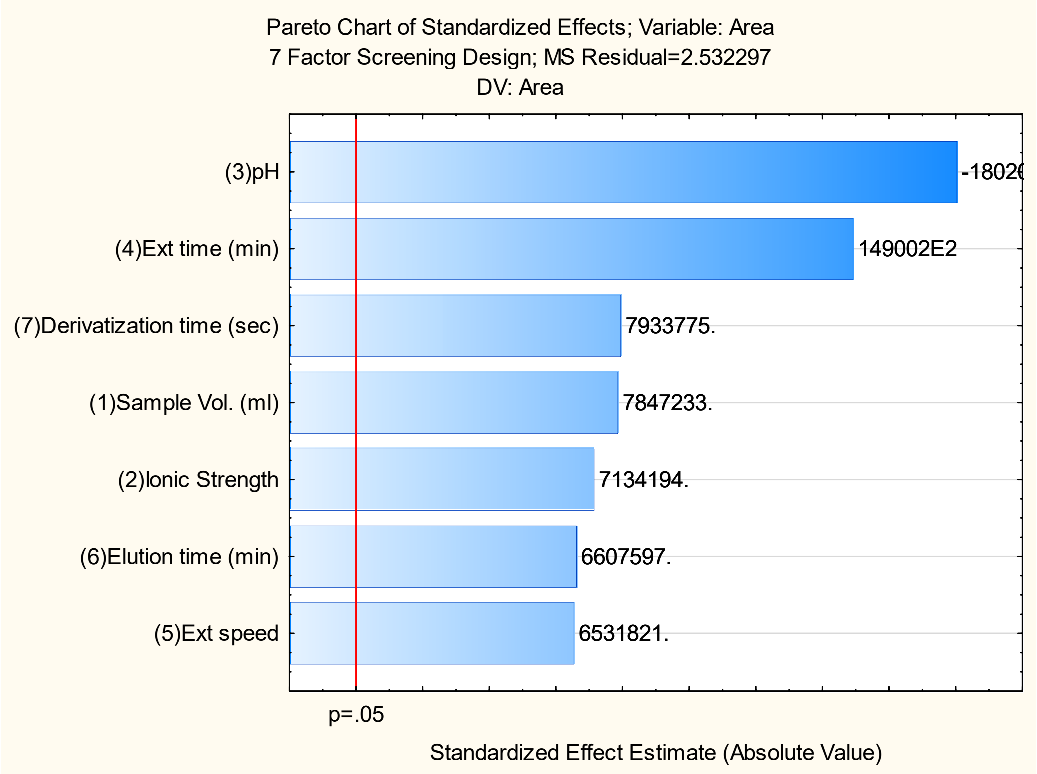

Figure S3. Pareto diagram of calculated main effect for PBD.

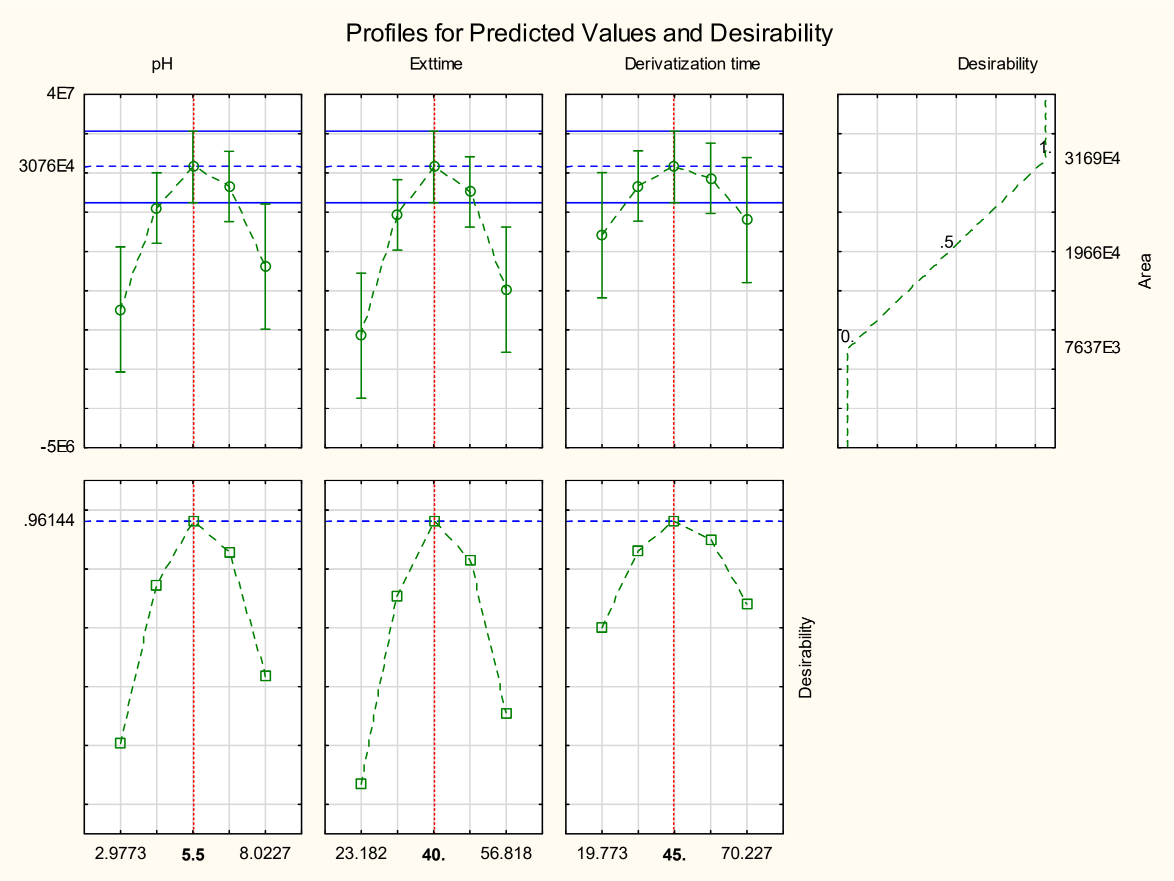

Figure S4. Desirability function plot showing the optimum range of significant factors.

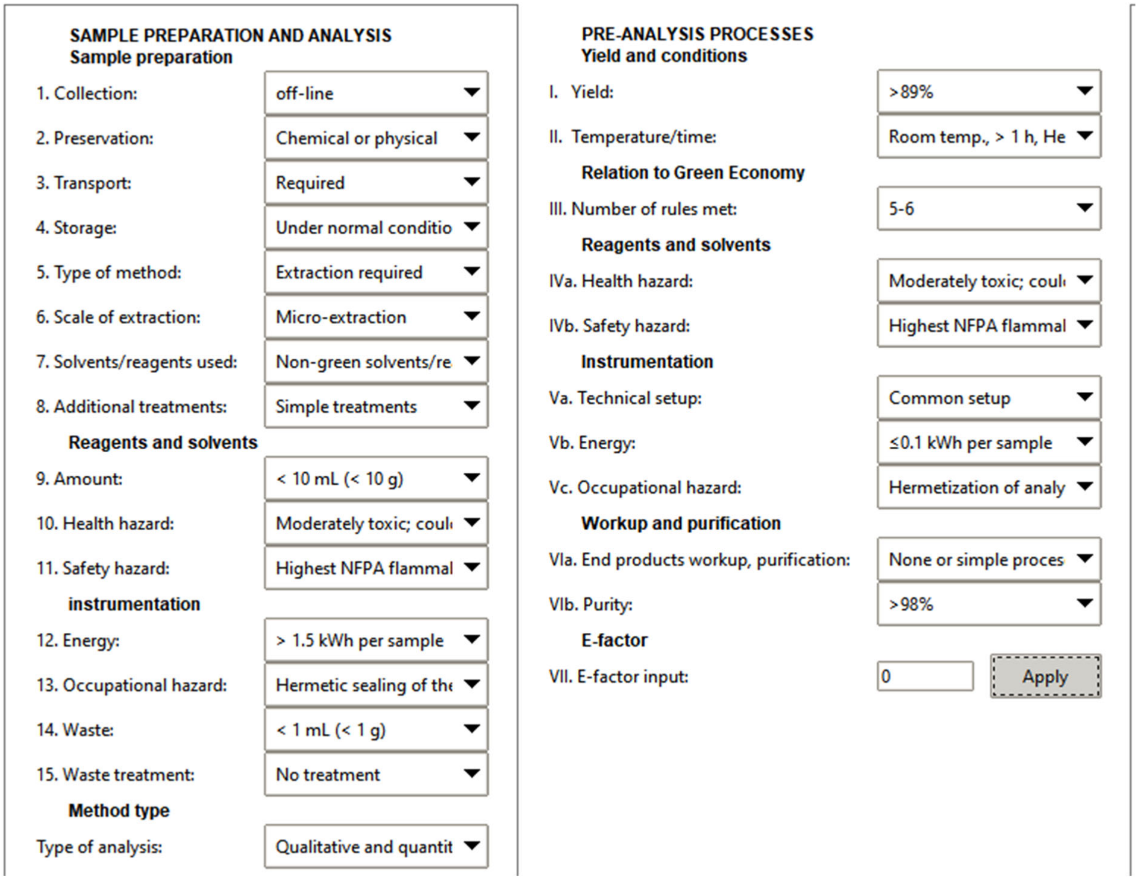

Figure S5. Input parameters and their values in ComplexGAPI software for the proposed method.
